# The Epigenetic Regulation of Blinatumomab Gene Expression: Tumor Cell-dependent T cell Response against Lymphoma Cells and Cytotoxic Activity

**DOI:** 10.22088/IJMCM.BUMS.8.1.55

**Published:** 2019-06-25

**Authors:** Fatemeh Naddafi, Fereidoun Mahboudi, Maryam Tabarzad, Zahra Aliabadi Farahani, Farshad Hosein. Shirazi, Fatemeh Davami

**Affiliations:** 1 *Pharmaceutical Sciences Research Center, Shahid Beheshti University of Medical Sciences, Tehran, Iran.*; 2 *Biotechnology Research Center, Pasteur Institute of Iran, Tehran, Iran.*; 3 *Protein Technology Research Center, Shahid Beheshti University of Medical Sciences, Tehran, Iran.*; 4 *Department of Biology, Science and Research Branch, Islamic Azad University, Tehran, Iran.*

**Keywords:** BiTE, T-cell activation, refractory acute lymphoid leukemia, therapeutic anti- CD19 mAb, Blinatumomab

## Abstract

Conventional treatment for cancer such as surgical resection and chemotherapy can cause damage in cases with advanced cancers. Moreover, the identification of tumor-specific targets has great importance in T-cell therapies. For decades, T cell activity has been stimulated to improve anti-tumor activity. Bispecific antibodies have attracted strong interest from pharmaceutical companies, for their diagnostic and therapeutic use. Blinatumomab is a first-in-class bispecific T engager antibody for the treatment of relapsed or refractory precursor B- cell acute lymphoblastic leukemia. But, it can benefit several cases with CD19^+^ malignancies in the future. PhiC31 integrase-based vectors could selectively integrate therapeutic transgenes into pseudo-attP sites in CHO genome. In this study, production of Blinatumomab in CHO cells using this type of vectors was investigated. We evaluated the effects of histone deacetylases (HDACs) inhibitors such as sodium butyrate and valproic acid, on specific productivity and cell viability of antibody expressing cells. Although sodium butyrate increased specific productivity about 1.7-fold and valproic acid about 1.4-fold, valproic acid was found more efficient because of its less cytotoxic effect on cell growth. We examined the efficacy of expressed Blinatumomab at various effector to target (E/T) ratios. A dose-response analyses of calcein-acetoxymethyl release assay illustrated that the effective dose of expressed mAb required for antibody mediated cytotoxicity was 100 ng/ml and the expressed mAb was more effective at E/T ratios of 10:1 and 5:1. Results of this study indicated that the expressed blinatumomab can be useful for enhancing the cytotoxicity of CD3^+^ T-cells against CD19 ^+^ target cells *in vitro*.

The phiC31 integrase mediates precise, unidirectional recombination between two attP and attB recognition sites (1). This serine integrase could integrate attB- containing donor plasmid into pseudo attP site in mammalian genomes (2). PhiC31 integrase system is considered as a specific tool for gene therapy  (3, 4) and transgenic research (2, 5). The efficiency of phiC31-integrase has been indicated to be comparable with that of the widely used Cre/loxP system. Furthermore, flippase (FLP) recombinase shows only 10% recombination activity on chromosomal targets in comparison with Cre recombinase (6). Cre and FLP cause deletion of the gene after integration (7) whereas phiC31 integrase can catalyze unidirectional and irreversible recombination between attB and pseudo attP sites (3). Development of phiC31 integrase-based vectors for prolonged therapeutic gene expression, demonstrated that it is a robust and reliable gene delivery system  (4, 8). Sodium butyrate (NaBut) treatment increases the specific productivity of recombinant proteins in mammalian cells; but, it declines cell growth and can provoke apoptosis  (9). NaBut inhibits the activity of many histone deacetylases, induces hyperacetylation of histones. Histone acetylation could modify chromatin structure, lead to transcription factors and polymerases binding as well as improving gene expression (10). Due to its impact on epigenetic mechanisms, NaBut has attracted many interest for the prevention and treatment of different diseases such as genetic/metabolic conditions and neurological degenerative disorders (11). Valproic acid (VPA), a histone deacetylase inhibitor (HDACi), can cause impaired epigenetic modification and suppress cell growth (12). It can increase the expression of genes that are regulated by transcription factors (13). It has been indicated that the HDACi increases both the specific productivity and mRNA transcription level in stable CHO cell lines. Furthermore, no cellular toxicity was reported with VPA compared with other widely used HDACi such as NaBut (14). Blinatumumab, the most advanced bispecific T-cell engager (BiTE) with dual binding specificities (15), was approved for precursor B-cell acute lymphoblastic leukemia (B-cell ALL) on December 3, 2014 (16). BiTE antibodies can form a transient cytolytic synapse between T cells and the tumor target cells. This leads to discharge of T cells contents and induces tumor cell death (17). Blinatumomab can redirect T cells toward malignant B cells, and induce cancer cell lysis. The 55 kDa bispecific antibody (BsAb) has an anti-CD3 arm to bind CD3+ T-cells, and an anti-CD19 arm to couple to CD19+ lymphoma cells (15). Preclinical studies illustrated that blinatumomab's efficacy is dependent on the effector-to-target ratio and on the difference between its affinity for both CD19 and CD3 antigens (18). In the present study, we investigated the phiC31 mediated gene integration for expression of a BiTE antibody (Blinatumomab) in CHO-DG44 cells. This is the first study in which phiC31 integrase is used for (BsAb) expression. We compared the effect of the HDAC inhibitors, NaBut and VPA on specific productivity and cell growth in stably transfected cells. The calcein-AM assay was used to determine the cytotoxic activity of the expressed monoclonal antibody (mAb).

## Materials and methods

**Cell lines and culture media**

CHO-DG44 cells suspension was obtained from Life Technologies, USA (Catalog no: A10971-01) supplemented with L-glutamine, PenSterp and anti clumping agent from Invitrogen (CA, USA). NALM-6 (CD19+) and Jurkat (CD3+) cell lines were purchased from ATCC. T-cell enrichment from PBMCs was performed using LYMPHOLYTE H CL5020 cell separation (Cedarlane Laboratories Ltd, Canada) according to the instructions of the manufacturer.

**Vector construction**

The Blinatumomab sequence was cloned into the FC550A-1 (System Biosciences, USA) plasmid by EcoRV restriction enzyme. Subsequently clones containing ligated plasmid were screened with XhoI restriction enzyme. All cloning steps were done according to our previous study (19). 6 × His-tag was introduced at the C-terminus of the construct for further detection and purification.

**Cell culture**

CHO-DG44 cells were grown and transfected in a chemically defined medium (Life Technolo-gies) by using X- tremeGENE HP transfection reagent (Roche, Mannheim, Germany). They were supplemented with 8 mM L-glutamine and 1% penicillin/ streptomycin (100 μg/ml) (Invitrogen, CA, USA) in disposable vented cap flasks, at 37 °C in humidified atmosphere of 5% CO_2_. Furthermore, the cells were sub-cultured every 3 days at a density of 3 × 10^5^ cells/ml. Cells density and viability were determined by using Trypan blue exclusion method.

X-tremeGENE HP was used to transfect the cells, based on manufacturer’s 6 well plate’s protocol. On the day of transfection, CD DG44 medium without any supplements and containing 2× 10^6^ cells/ml was seeded in 6 well plates. In order to optimize, different ratios of µL X-tremeGENE HP to µg DNA have been tested. The transfection complex was formed at a DNA: X-tremeGENE HP ratio of 3:9 and diluted in 300 μl of serum-free media (SFM) (Invitrogen, CA, USA). After incubation at room temperature for 30 min, they were added drop wise to the cells.

**Generation of stable cell pools**

**Fig. 1:**
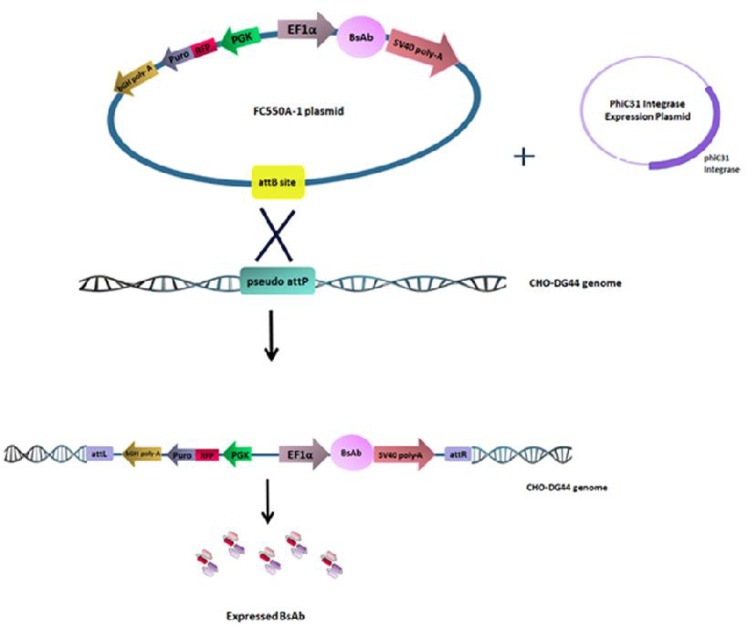
**Schematic model of the FC550A-1 as a donor plasmid.** As well as, the role of phiC31 integrase in recombination of donor plasmid sequence into pseudo-attP sites in CHO genome was demonstrated . The integrated gene of BsAb was stably expressed

The cells were transfected in duplicates as mentioned in the previous section. Then, cells were counted and co-transfected with 3 μg of FC550A-1 donor vector (containing BsAb gene) and 1.5 μg of the helper vector (which was reported in another study) using X-treme DNA Gene HP transfe-ction reagent  (20). FC200A-1 vector can transiently express the phiC31 integrase in order to insert the donor expression vectors into pseudo attP genomic sites by using the attB sequences in the FC550A-1 donor vectors. Duplicate test conditions were incubated with the prepared reagent/ DNA mix at 37 °C, 5% CO_2_ for 48 h. The control group was FC550A-1 donor vector without helper vector. Transfected cells were selected by using CDDG44 medium containing varying concentrations of puromycin (2, 4, 6, 8, 10 μg/ml). Cells were maintained under puromycin selection until the cell viability reached to more than 90% after 21 days of selection. Throughout the selection period, cells were passed in a fresh medium twice per week. Cells were scaled up using 25 and 75 cm^3^ flasks (Greiner, Belgium). The determined minimum inhibitory concentration (MIC) result for stable pool production was 4 μg/ml (Fig 1).

**Antibody purification**

Based on our previous study, supernatants of the stable cell pools containing the expressed BsAb were collected to be purified by using nickel nitrilotriacetic acid (Ni-NTA) resin (QIAGEN, USA). (19). The fraction containing the expressed antibody were collected in 1-ml tubes and stored at –20 °C.

**SDS-PAGE and western blot analysis**

The expressed mAb in protein level was analyzed by SDS-PAGE and western blotting. SDS-PAGE was performed on 12% polyacrylamide gels. The bands were appeared by means of coomassie brilliant blue staining. For western blotting, the purified supernatants were run on polyacrylamide gel and transferred onto nitrocellulose membrane (GE Healthcare, USA) by using a Trans-Blot SD semidry transfer cell (BioRad, Hercules, CA, USA). The bands were detected by 3,30-diaminobenzidine (DAB) (Sigma-Aldrich, Missouri, USA).

**Binding activity of expressed BsAb**

We used In-Cell ELISA to study the recognition of both CD3 and CD19 antigens by expressed BsAb. NALM-6 and Jurkat cell lines were utilized. A CD19/CD3 negative cell line (SK-BR-3 cell line) was used as control group. The optical density (OD) of the reactions was measured at 450 nm for each well using an ELISA plate reader (Bio-Rad, Hercules, CA) (19, 21).

**Determination of specific productivity**

To calculate the specific productivity of cells, the cell pool of expressed mAb was cultured. 2×10^5^ cells were seeded in shaker bottles, and viable cell density and antibody titer were evaluated during five days. Viable cell density was determined using Trypan blue exclusion method. 

Specific productivity (Qp) was measured in pg/cell/day by using the following equation  (22):

$\mathrm{Qp}=\frac{10\mathrm{.in}(\frac{n_{t}}{n_{0}})}{(n_{t}-n_{0})t}$

Where ΔP shows the change in antibody titers (μg/ml) between the first and last days of the test, n_0_ and n_t_ indicate viable cell densities (10^6^ cells/ml) in the first and last days, and t shows culture time in days.

**Sodium butyrate and valproic acid treatment**

To evaluate the effect of NaBut and VPA on BsAb productivity, 1 × 10^6^ cell/ml were cultivated in 6-well tissue culture plates in Pro CHO5 medium (Lonza, Basel, Switzerland) supplemented with 100 mM hypoxanthine, 16 mM thymidine (Sigma–Aldrich, Missouri, USA), and 8 mM glutamine and grown under shaking at 125 rpm. The successfully transfected CHO cells were grown in the presence and absence of NaBut / VPA. Stocks of 1 M NaBut and 500 mM VPA (Sigma–Aldrich, Missouri, USA) were prepared in PBS and sterilized with a 0.22- μm filter (23). Different concentrations of NaBut (1, 1.5, 2 mM) and VPA (100, 250, 500, 1000 μM) were tested. The optimized concentrations of NaBut and VPA were 2 mM and 500 μM, respectively. The antibody yields were determined after 5 days of treatment (23, 24).

**Extreme cytotoxic efficacy of BsAb with unstimulated human T lymphocytes**

A fluorescence-based cytotoxicity assay was performed using the fluorochrome calcein-acetoxymethyl (calcein-AM) dye (R&D Systems, USA). It is trapped inside live target cells and can only release upon redirected lysis. T-cell enrichment from peripheral blood mononuclar cells (PBMCs) of healthy donors (according to the ethical and legal issues in research involving human subjects, Ethical approval code of project: SBMU. REC. 1393. 642) was performed according to the instructions of the manufacturer (LYMPHOLYTE H CL5020 cell separation (Cedarlane Laboratories Ltd, Canada)). Then, they were centrifuged (800 × g, 10 min) and washed twice with RPMI-1640 medium. The supernatant was discarded and isolated PBMCs were collected and cultured in RPMI 1640 medium supplemented with 10 % (v/v) FBS. The cells were counted using trypan blue and a hemacytometer. Freshly prepared PBMCs (300,000 cells/well) were added to each well of a black-walled microplate. NALM-6 cells were grown in RPMI 1640 supplemented with 10% FBS, washed with 1X calcein AM DW Buffer, and counted using trypan blue and a hemocytometer. Then, incubated with 1 μM calcein AM for 30 min at 37 °C under 5% CO_2_. Finally, 30,000 cells/well were added to each well of a black-walled microplate. T cells from PBMCs of healthy donors were mixed at a ratio of 10:1, 5:1, 2.5:1 with calcein AM labeled NALM-6 B lymphoma cells. Moreover, the concentrations of the BsAb were 0.001, 0.01, 0.1, 1, 10, 100 and 1000 ng/ml. After 4 h, calcein AM was fluorometrically determined in the culture medium. Fluorescence values were recorded using a 490 nm excitation filter and a 520 nm emission filter. Moreover, these data were compared with the fluorescence signal from a control reaction with no cytotoxic compound and a reaction in which the fluorescence signal was determined for totally lysed cells in 1% saponin (Sigma–Aldrich, Missouri, USA) (for 10 min). The specific cytotoxicity was calculated by the equation below

Specific cytotoxicity=[fluorecence sample-fluorecence (control)][fluorecence total lysis-fluorecence (control)]×100

**Statistical analysis**

Obtained data were statistically analyzed in GraphPad Prism 6 software using the one-way ANOVA and t test (P value ≤ 0.05). That is to say, calcein-AM release assay was performed as technical triplicates and ELISA assay was performed in duplicates.

## Results

**Construction of the expression plasmid**

The bispecific monoclonal antibody, Blinat-umomab sequence was successfully cloned into the EcoRVsite of FC550A-1 vector. 

**Generation of stable cell pools using phiC31 integrase system**

**Fig. 2:**
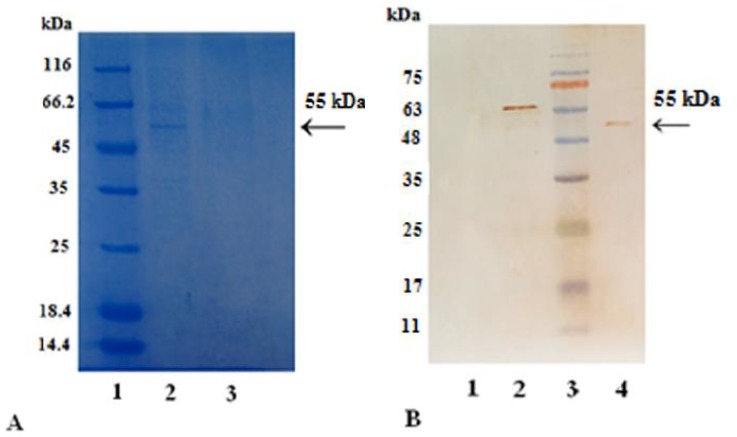
**SDS-PAGE and western blot analysis. **A: SDS-PAGE analysis of a purified mAb, blinatumomab. The band related to BsAb expressed by CHO- DG44 cells is shown by the arrow. Lane 1: protein molecular weight marker (14.4- 116 kDa); lane 2: CHO-DG44 cells supernatant transfected with FC550A-1 vector; lane 3: un-transfected CHO cells supernatant (negative control). B: western blot analysis of expressed protein in CHO cells. Lane 1: un-transfected CHO cells supernatant (negative control); lane 2: purified UreB – His tag protein (63 kDa) as positive contro; lane 3: pre-stained protein marker (14.4- 116 kDa); lane 4: CHO-DG44 cells supernatant transfected with FC550A-1 vector (55 kDa)

**Fig. 3:**
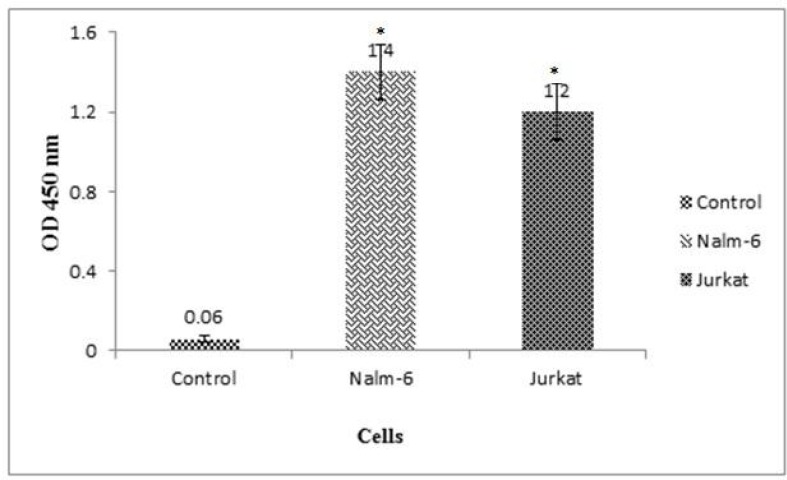
**The binding affinity of expressed BsAb to CD3 and CD19 antigens.** Two NALM-6 and Jurkat cell lines had been used. A CD19/CD3 negative cell line (SK-BR-3 cell line) was used as control group. Error bars indicated standard deviation of duplicate measurements. Differences between BsAb binding to NALM-6 and Jurkat cell lines were statistically significant in comparison with control cell line (P<0.05)

In this research, we used the robust phiC31 dual vector system for the construction of stable CHO-DG44 cell pool. During antibiotic selection, cell pools without phiC31 integrase expression vector showed random integration which could not resist antibiotic selection. This might be due to lack of sufficient phiC31 donor vector efficiency to integrate plasmid DNA into hot spots in the genome. Following induction and transfection, purification of BsAb from the supernatant of transfected CHO-DG44 cells yielded 0.5 mg/l. Purified supernatant of the transfected cells with both phiC31 integrase expression and donor vector, were tested for BsAb expression by SDS-PAGE and western blotting (Fig 2). The specific bound of the purified protein was observed at 55 kDa. The binding activity of purified antibody to both CD3 and CD19 was tested using In-Cell ELISA which is presented in Fig 3.

**Effect of sodium butyrate and valproic acid on protein expression in CHO-DG44 suspension cells**

**Fig. 4:**
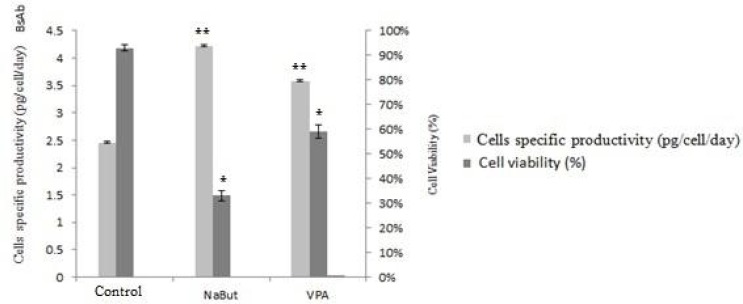
**Influence of NaBut and VPA on cells specific productivity and cell viability.** Stable BsAb producer cell line was cultured in the absence and presence of NaBut and VPA. Error bars indicated SD of triplicate measurements.* indicates that the differences of cell viability of NaBut and VPA treatment group were significant in comparison with control group (P < 0.05). ** indicates that the differences of cell specific productivity of NaBut and VPA treatment groups were significant in comparison with control group (P < 0.05). The difference between cell viability of NaBut treatment group and VPA treatment group was also significant (P < 0.05)

NaBut had growth-inhibiting effects on transfected cells. It declined cell viability over the control group (BsAb producer untreated cells).. Moreover, NaBut enhanced the specific cell productivity (4.22 pg/cell/day) more than VPA (3.58 pg/cell/day). But, the cell viability observed after NaBut treatment was lower than that of VPA. Thus, treatment of cells with NaBut and VPA enhanced specific productivity about 1.7-fold in NaBut-treated cells and 1.4-fold in VPA -treated cells (compared with untreated cells (2.46 pg/cell/day)). NaBut treatment decreased the cell viability by 33% in comparison with control group (P<0.05), while VPA-treated cells viability was 59% which is significantly higher than NaBut-treated cells (P<0.05) (Fig 4).

**Calcein-AM release assay**

**Fig. 5:**
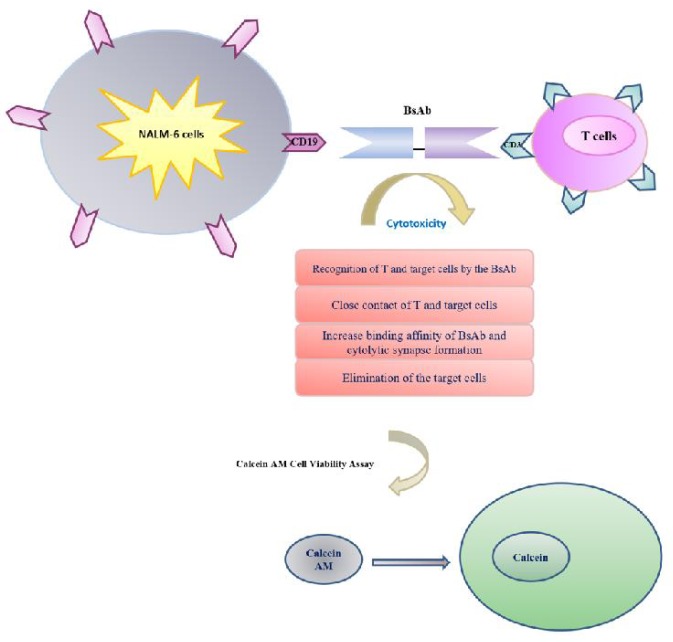
**Schematic of tumor cells killing by cytotoxic T cells redirected with an expressed BsAb (Blinatumomab).** 4 phases were involved. The calcein AM assay was used to quantify cell viability and cytotoxicity

**Fig. 6:**
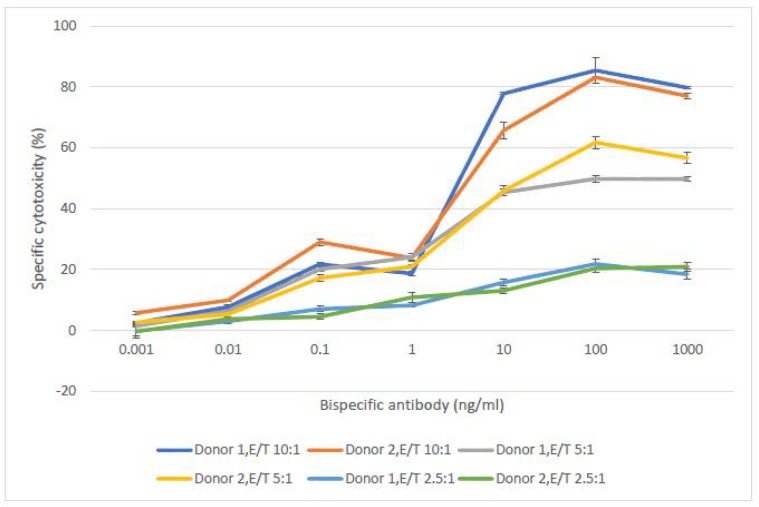
**Dose-response curve of expressed mAb.** In this experiment PBMCs from 2 donors were used. Purified T cells were incubated with NALM-6 cells at E/T ratios of 10:1, 5:1, 2.5:1 and cell lysis was determined by calcein AM release after 4 h. The test was done in triplicate. The error bars indicate the standard deviation of two groups. The cytotoxicity observed at higher ratios than 2.5:1 was significantly higher at the concentrations higher than 1 ng/ml (P < 0.05)

We tested the efficacy of BsAb at effector to target (E/T) ratios ranging from 10:1 to 2.5:1 by using purified, un-stimulated T lymphocytes as effectors and NALM-6 cells as target cells (Fig 5). As illustrated in Fig 6, E/T ratios of 10:1 and 5:1 had very similar dose-response curves (P > 0.05). Furthermore, the percentage of speciﬁc cytotoxicity was almost similar at E/T ratios of 10:1 and 5:1. But, it was decreased at an E/T ratio of 2.5:1. The results proved that the BsAb can be effective at low E/T ratios too. The percentage of speciﬁc cytotoxicity varied from 21 to 87%. The specific cytotoxicity percentage was determined to be 87.48%, 61.14% at E/T ratios of 10:1 and 5:1, respectively. While, at an E/T ratio of 2.5:1 the extent of speciﬁc cytotoxicity decreased to 21.14%. Thus, at E/T ratios of 10:1 and 5:1, the expressed BsAb was significantly more cytotoxic than 2.5:1 (P<0.05). Dose-response curves with 2 selected healthy donor T cells are shown in Fig 6. The extent of speciﬁc cytotoxicity during the 4 h assay period obtained with T-effector cells from 2 PBMC donors indicated no considerable variation among the two PBMC donors. The data proved that the expressed BsAb could be effective at low E/T ratios, too. A dose-response analysis of calcein AM release assay showed that the effective dose of expressed mAb required for antibody mediated cytotoxicity was 100 ng/ ml.

## Discussion

The phiC31 integrase is considered as a non-viral site-specific gene therapy vector. In comparison with viral vectors, non-viral vectors could be safer with no immunogenicity  (4). It has been shown that plasmid backbone sequences have a crucial role in integration efficiency, transgene persistence as well as duration of transgene expression from episomal vector DNA (25). Non-viral gene therapy has become an approach for cancer therapy. More than 17% of all gene therapy trials used non-viral vectors. Therefore, non-viral vectors can be as an alternative medicine to viral vectors for the proper expression and therapeutic genes delivery (26). It has been proved that in the presence of the phiC31 integrase, a plasmid containing attB is efficiently integrated into mammalian genomes. Moreover, about 90% of the integration events are integrase-mediated in unmodified human cells, and distributed among a set of pseudo attP sites (27, 28). In this study, we co-transfected CHO-DG44 cells with a plasmid containing the puromycin resistance marker and attB site (FC550A-1), with and without a plasmid expressing the phiC31 integrase (FC200A-1). Our results confirmed that FC200A-1 vector plays an important role in efficient site-specific genomic integration in CHO cells, since the cells without this vector showed non- resistant random integration.

In order to bring down the costs of recombi-nant proteins production in the biopharmaceutical industry, researchers worked on regulatory principals of growth and survival during the cell culture process (29). One strategy for increasing CHO cell productivity is the addition of HDAC inhibitors, NaBut, and VPA (29, 30). Although, the specific productivity of NaBut was slightly more than VPA, the decrease in cell viability was remarkable in NaBut-treated group. Since VPA is an FDA-approved drug and 5-fold less expensive than NaBut, it is suggested as a cost-effective alternative to NaBut for increasing protein expression in CHO cells (24). NaBut has been demonstrated to increase the expression of genes controlled by mammalian promoters such as cytomegalovirus (CMV) and simian virus 40 (SV40)    (31), but it could decrease cell growth and lead to cellular apoptosis    (32). In one study, VPA induced cell cycle arrest at G1 phase and was considered as an effective chemical reagent to enhance mAb production in recombinant CHO cells (33). Here the effects of two HDAC inhibitors on stable gene expression using CHO-DG44 cells as host were investigated. Although NaBut increases the specific productivity more than VPA, VPA showed less cytotoxic effects on CHO cells. Therefore, VPA could be a more efficient alternative to NaBut in CHO-DG44 cells.

Our findings proved that the expressed BsAb induced cytotoxicity of T-cells against NALM-6, a CD19+ cell line, *in vitro*. We tested the efficacy of expressed bsCD19×CD3 at E/T ratios ranging from 10:1 to 2.5:1 using PBMC of 2 healthy donors as effectors and NALM-6 cells as target cells. As illustrated in Fig 6, E/T ratios from 10:1 to 5:1 indicated very similar dose-response curves. Several studies confirmed the induction of the cytotoxicity of effector cells against target tumor cells by using BsAbs (-). In one study, it has been shown that bsCD19×CD3 redirected un-stimulated cytotoxic T cells against CD19^+^ cells in an unexpectedly potent, rapid form      (39). In another study, a fully human recombinant BsAb that targeted both CD3 and CD19 was as a powerful stimulator of T cell proliferation. In the presence of un-stimulated T cells, it could induce specific cytotoxicity against non-Hodgkin’s lymphoma Raji cells (40). The expressed BsAb showed the dependence of efficacy upon E/T ratio over the range assayed as it was less effective at low E/T ratios. It was reported that T cells undergo multiple rounds of target cell elimination in the presence of the BsAb. Moreover, the effective dose of expressed mAb for antibody mediated cytotoxicity was 100 ng/ml. The dependency of BiTE-mediated cytotoxicity on both target expression and E/T ratio had also been demonstrated in previous studies (39, -). It has been indicated that the bispecific CD19/CD3 antibody had significant cytotoxic activity at concentrations of 10 to 100 pg/ml and at E/ T ratio of 2:1 (45).

BsAbs have attracted much interest for their specificity, cost, and ease of production. We used phiC31 integrase as an efficient, site-specific, unidirectional integration system for BsAb expression in CHO cells. Our findings confirm that addition of NaBut and VPA to CHO cells stably expressing BsAb, could increase specific productivity. But, VPA was observed to be more efficient and cost effective when compared to NaBut. Moreover, the obtained results of this study demonstrated that the expressed blinatumomab can be useful for enhancing the cytotoxicity of CD3^+^ T-cells against CD19 ^+^ target cells *in vitro*. Its efficacy depended on E/T ratio, since it was found to be less effective at low E/T ratios.

## Conflict of interest

Authors declare no conflict of interest
